# Interaction-induced zero-energy pinning and quantum dot formation in Majorana nanowires

**DOI:** 10.3762/bjnano.9.203

**Published:** 2018-08-15

**Authors:** Samuel D Escribano, Alfredo Levy Yeyati, Elsa Prada

**Affiliations:** 1 Departamento de Física de la Materia Condensada C3, Universidad Autónoma de Madrid, E-28049 Madrid, Spain; 2 Departamento de Física Teórica de la Materia Condensada C5, Condensed Matter Physics Center (IFIMAC) and Instituto Nicolás Cabrera, Universidad Autónoma de Madrid, E-28049 Madrid, Spain

**Keywords:** hybrid superconductor–semiconductor nanowires, interactions, Majorana bound states, quantum dots

## Abstract

Majorana modes emerge in non-trivial topological phases at the edges of specific materials such as proximitized semiconducting nanowires under an external magnetic field. Ideally, they are non-local states that are charge-neutral superpositions of electrons and holes. However, in nanowires of realistic length their wave functions overlap and acquire a finite charge that makes them susceptible to interactions, specifically with the image charges that arise in the electrostatic environment. Considering a realistic three-dimensional model of the dielectric surroundings, here we show that, under certain circumstances, these interactions lead to a suppression of the Majorana oscillations predicted by simpler theoretical models, and to the formation of low-energy quantum-dot states that interact with the Majorana modes. Both features are observed in recent experiments on the detection of Majoranas and could thus help to properly characterize them.

## Introduction

Semiconducting nanowires with strong spin–orbit interaction, such as InAs or InSb, are becoming ideal systems for the artificial generation of topological superconductivity [1–3]. In addition to its fundamental interest, such nanowires that may host Majorana bound states (MBSs) at their ends or interfaces [4–5] constitute promising platforms for Majorana-based quantum computing devices [6–9]. Progress in fabrication techniques has allowed to induce a hard superconducting gap in InAs [10] or InSb [11] nanowires with epitaxially deposited Al layer. Moreover, last-generation devices exhibit a very low degree of disorder, which allows them to almost reach the ballistic limit [12–14].

In spite of these advances, the experimental signatures of MBSs in the nanowire devices deviate significantly in several aspects from the theoretical predictions of minimal models. This is the case, for instance, regarding the behavior of the subgap conductance through the proximitized nanowire, which has been addressed in several experiments [10,12–19]. In a long wire (the length of which is much greater than the induced coherence length) the presence of MBSs manifests itself in the appearance of a zero-bias conductance peak the width of which is controlled by the normal-state conductance [20]. However, for typical wire lengths explored in actual experiments, which are of the order of a few micrometers, it is expected that the overlap between MBSs located at both ends of the wire gives rise to conventional Andreev bound states that deviate from zero energy, leading to an oscillatory pattern in the low-bias conductance as a function of Zeeman field, chemical potential or wire length [21–23]. Conspicuously, in most of the available experimental data the emergence of a robust zero-bias conductance peak is observed above some critical Zeeman field without the expected oscillatory pattern [12,19,24–25]. Several mechanisms have been proposed to account for the reduction or lack of oscillations, such as smooth confinement [21,26–28], strong spin–orbit coupling [29], position-dependent pairing [30], orbital magnetic effects [31], Coulomb repulsion among the carriers in the nanowire [22], or the presence of the normal drain lead connected to the hybrid wire [32].

Another source of Majorana oscillation suppression was put forward by some of us in a recent work [33]. The key realization is that MBSs in a finite-length wire posses a finite charge, typically distributed uniformly along the wire [34], which can be susceptible to electrostatic interactions with the surrounding medium. We considered the case of a grounded parent superconductor, thus avoiding the effect of a charging energy associated to the Cooper pairs, and showed that, in such case, a residual effect of interactions may arise from the image charges induced in the electrostatic environment of the nanowire. Using a simple model for the induced potential we concluded that, in typical experimental setups, interactions would lead to pinning of the MBSs to zero energy around parity crossings and, thus, to more robust zero-bias conductance peaks than predicted by the non-interacting models.

**Figure 1:**
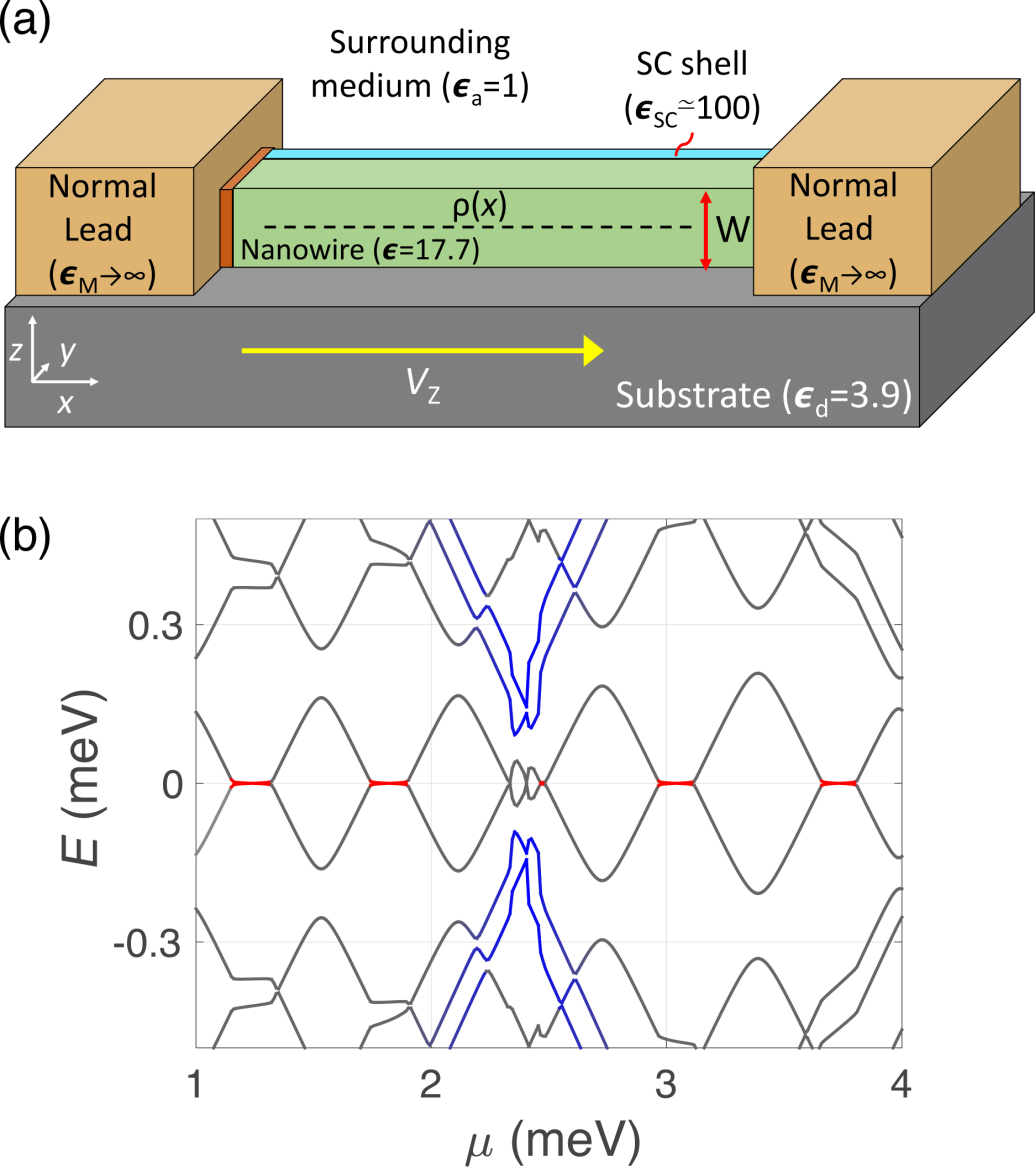
(a) Schematic representation of the setup analyzed in the present work. A nanowire of rectangular cross section (green) lying on an insulating substrate (grey) and in contact to a thin metallic layer in one of its faces (light blue), corresponding to the parent superconductor, and two normal metal leads at its ends (orange) separated by tunnel barriers (brown). Typical values for the dielectric constants for each region are indicated. (b) Low-energy spectrum as a function of the chemical potential μ for a wire of thickness *W* = 100 nm and length *L* = 1 μm. Other parameters are the spin–orbit coupling α = 20 nm·meV, the induced pairing energy Δ = 0.3 meV and the Zeeman energy *V*_Z_ = 2 meV. Electrostatic environment-induced zero-energy pinned regions between Majorana oscillations are indicated in red. Quantum-dot levels (in blue), occurring at the edges of the wire due to the interaction with the bulk contacts, anticross with Majorana levels and remove their zero-energy pinning.

The aim of the present work is to test the validity of the predictions of [33] for the case of more realistic calculations of the induced electrostatic potential, taking into account the actual three-dimensional (3D) geometry as well as the effect of nearby metallic leads. We consider the geometry depicted in Figure 1a, where a nanowire of rectangular cross section lies on an insulating substrate (typically SiO_2_) and is contacted to a thin superconducting (SC) layer on one of its faces and to two bulk normal leads at both ends, separated by thin insulating barriers. In Figure 1a we indicate the characteristic dielectric constants of each region, which are relevant for the calculation of the induced potential through Poisson’s equation (discussed below). Our aim is to solve this equation together with the Bogoliubov–de Gennes equation for determining self-consistently the charge density ρ(*x*) along the nanowire. For this purpose we derive a generalized method of image charges that allows us to calculate the induced potential under rather general conditions, taking into account a 3D electrostatic environment as the one shown in Figure 1a.

We find two main effects coming from this interaction, which are exemplified in Figure 1b. One is, as stated before, the suppression of Majorana oscillations around parity crossings (zero-energy crossings where the total fermion parity of the wire changes), both as a function of the Zeeman energy *V*_Z_ and the chemical potential μ of the wire. This effect is produced because, at each parity crossing, a finite Majorana charge *Q*_M_ enters the wire from the reservoir in an abrupt fashion. If the electrostatic screening is smaller inside the wire than in the reservoirs, a repulsive interaction is produced between the incoming charge and its images, preventing its entrance. This translates into finite regions in parameter space (in red in Figure 1b) where Majorana modes are pinned to zero energy within a finite range of *V*_Z_ or μ proportional to the Majorana charge *Q*_M_ and the strength of the interaction. This was already shown in [33] but for a simplified dielectric profile where the presence of the superconducting shell had been ignored. We here include it and find that the size of the pinned regions decreases but the pinning effect is still present under certain conditions that we discuss in detail below. Moreover, we explain the incompressible behavior of the electron liquid within these pinned regions in terms of the Majorana wave functions and their charge.

Another important effect of the electrostatic environment unexplored before is the creation of deep potential wells at the ends of the wire close to the bulk metallic electrodes. These wells, obtained explicitly here through the self-consistent calculation, are similar to the confinement potentials typical of quantum dots. Localized quantum dot-like energy levels in these regions disperse with magnetic field (or chemical potential) and appear below the induced gap in the wire spectrum (in blue in Figure 1b). In the topological regime, dot-like levels interact with Majorana states, anticrossing them when they approach zero energy. Similar phenomena were observed in some experiments [14,19], and have been likely found on other occasions but discarded by experimentalists looking for the simpler picture. Interestingly, it has been shown that the shape of these anticrossings can be used to quantify the degree of non-locality of the Majorana wave functions [35–36], a prediction that has been experimentally demonstrated recently [25]. Here, we show that if the interaction between dots and Majorana levels occurs in a pinning region, Majorana levels are forced to depart from zero energy, revealing the existence of a finite wave function overlap between them in spite of their zero energy. We analyze this behavior again in terms of the wave functions of Majorana state and dot and their charge.

The paper is organized as follows: in the following section we provide insight into the theoretical model used to treat interactions. In the next section we analyze the case in which the influence of the bulk normal leads can be neglected, recovering the pinning effect found in [33] for a repulsive electrostatic environment. However, we focus here on the electrostatic environment effects on the Majorana wave function, rather than on its spectral properties. In the next section we study the effect of including the bulk normal leads of Figure 1a, finding that they give rise to the formation of quantum dot-like bound states. We further analyze the interplay of such states with the MBSs. Finally, we present the conclusions of our work. The robustness of the pinning effect is analyzed in detail in Section 4 of Supporting Information File 1.

## Model and Theoretical Approach

We model the electronic states along the proximitized Rashba nanowire of length *L* using the following single-channel Hamiltonian [4–5]

[1]
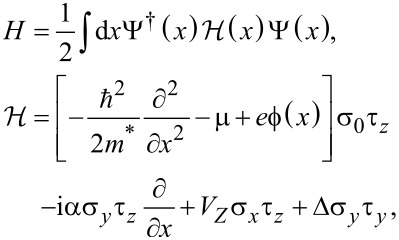


where 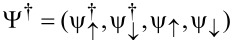 is a Nambu bi-spinor, ψ_↑_*_,_*_↓_(*x*) are electron annihilation operators, and σ and τ are the Pauli matrices in spin and Nambu space, respectively. The model is defined by setting the parameters m*, μ, α, *V*_Z_ and Δ, corresponding to the effective mass, the chemical potential, the spin–orbit coupling, the Zeeman energy caused by an external magnetic field, and the induced SC pairing potential, respectively.

In Equation 1, we also include the electrostatic potential 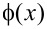 felt by charges in the nanowire, which can be decomposed as 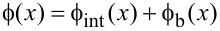, where 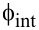 is the potential that arises from the free charges inside the nanowire, while 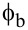 corresponds to the potential created by bound charges that emerge in the electrostatic environment. We compute the electrostatic potential using Poisson’s equation

[2]
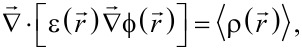


where 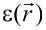 is the non-homogeneous dielectrical permittivity of the entire system and 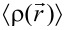 is the quantum and thermal average of the charge density of the nanowire obtained with Equation 1. The intrinsic part 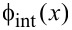 of the potential satisfies an analogous equation with a uniform ε equal to that of the nanowire. The geometry depicted in Figure 1a is taken into account through a piecewise 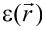 function where each material is characterized by a different dielectric constant, so that 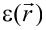 changes abruptly at the interfaces. Then, assuming that the charge density in the nanowire is located along its symmetry axis (*x*-axis), we obtain the electrostatic potential 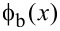 using the method of image charges, as explained in detail in Section 1 of Supporting Information File 1. More precisely, 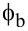 is given by


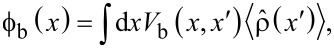


where *V*_b_(*x*,*x*^′^) is a kernel determined in order to satisfy the proper boundary conditions. We find analytical expressions for *V*_b_(*x*,*x*^′^). They are simple but rather lengthy and are given in Supporting Information File 1 for two different cases: neglecting the effect of the bulk normal leads at the wire ends and including it. The results for these two cases are analyzed in the following sections.

The obtained potential 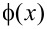 on the nanowire axis should be plugged back into Equation 1. The combined Poisson–Schrödinger problem must then be iterated until it achieves self-consistency. As shown in [33], the 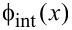 part of the electrostatic solution (i.e., the intrinsic electron–electron interaction part of the problem), treated at the Hartree–Fock level, has a negligible effect on the low-energy spectrum in the topological regime. We may therefore concentrate only on the self-consistency with 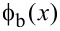. In Section 2 of Supporting Information File 1 we explain in detail the self-consistent numerical method used to compute the electrostatic potential profile as well as the eigenvalues and eigenvectors of Equation 1. For completeness, in Section 3 of Supporting Information File 1 we also show the effect of including the intrinsic interaction from 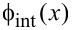, proving that its effect is small and that the main contribution stems from 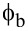.

In the following calculations, we consider the dielectric constants shown in Figure 1a. For the dielectrics materials, i.e., the wire, the substrate and the surrounding medium, we use typical values [37] of ε = 17.7, ε_d_ = 3.9 and ε_a_ ≈ 1, respectively. For the metallic leads we assume that, because they are bulky, they screen external electric fields perfectly, i.e., ε_M_→∞). This may not be the case for the SC shell, the capability of which for screening external electric fields may be weaker due to its small thickness and the unavoidable presence of disorder [38]. If this is the case, it is then characterized by a finite effective dielectric permittivity which depends on the SC shell width as well as its composition, as we show in Section 1 of Supporting Information File 1. Some experiments [39] have reported that for ultrathin metallic layers (ca. 5–10 nm) it is of the order of ε_SC_ ≈ 100. For these values, as we show in the next section, we find a repulsive environment, i.e., an environment the effective permittivity of which is smaller than that of the wire so that the bound charges that arise at the interfaces have on average the same sign as the free charges. We consider in Section 4 of Supporting Information File 1 the generality of our results as a function of the dielectric constant of the SC and the location of the charge density within the nanowire section. Below, in Figure 4c we show that, when the charge density is fixed at the center of the wire, as ε_SC_ becomes larger the dielectric environment turns into an attractive one and the pinning effect is eventually lost. This, however, strongly depends on the location of the charge density. If, as pointed out in [40], it happens to be close to the SC shell, the screening effect is larger and the pinning is suppressed. Nevertheless, as we analyze below in Figure 4e, even if ε_SC_→∞, the pinning effect remains when the wave function is located further away from the SC.

## Results and Discussion

### Results without bulk normal leads

**Figure 2:**
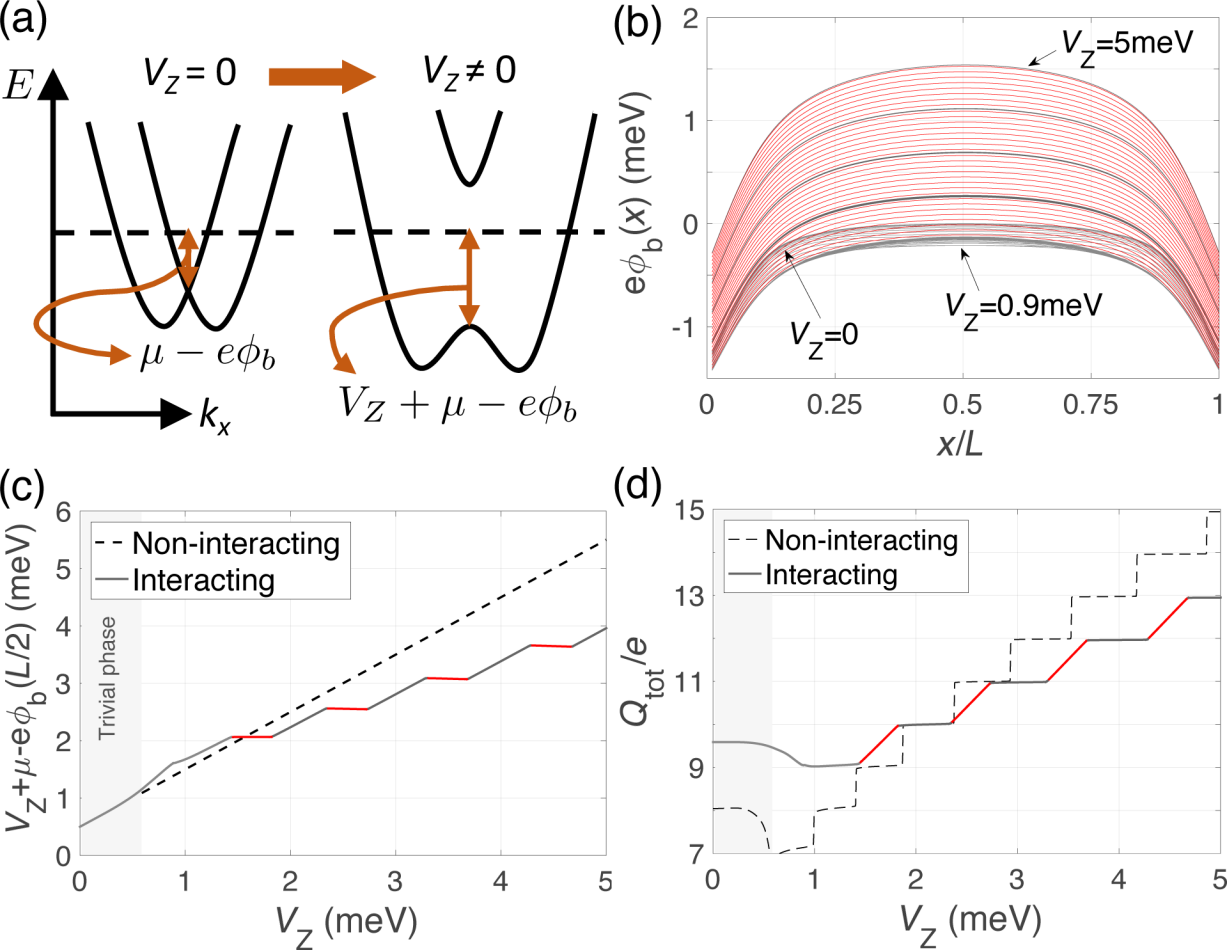
Majorana nanowire subject to interactions from the electrostatic environment (ignoring the influence of the bulk normal leads at its ends). (a) Schematic of the dispersion relation of the nanowire in the absence and in the presence of a Zeeman field. (b) Self-consistent induced potential energy 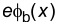 along the length of the wire for increasing values of the Zeeman splitting. Wire parameters as in Figure 1b and with μ = 0.5 meV. (c) Energy difference between the Fermi level and the band bottom at the center of the nanowire, *V*_Z_ + μ − 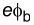
(*L*/2), and (d) total charge *Q*_tot_ of the nanowire as a function of *V*_Z_ for the non-interacting (dashed) and interacting (solid line) cases. Red curves highlight parameter regions for which there is interaction-induced zero-energy pinning in the spectrum.

It is convenient to start by analyzing the simpler case in which we neglect the effect of the bulk normal leads in the induced potential 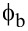. As an example we consider a nanowire of width *W* = 100 nm, length *L* = 1 μm and the following choice of realistic parameters: m* = 0.015*m*_e_, α = 20 nm·meV, Δ = 0.3 meV, μ = 0.5 meV and *T* = 10 mK. These could correspond, for example, to an InSb nanowire in contact to an Al superconducting shell [14], but similar results are obtained for InAs wire parameters [19]. For an infinite wire, a schematic representation of the energy bands is shown in Figure 2a in the absence and in the presence of a Zeeman field. At zero temperature, the occupied states below the Fermi level are those between the horizontal dashed line and the band bottom. Apart from a small contribution coming from the spin–orbit energy, the position of the band bottom is controlled by the chemical potential of the wire μ, the Zeeman energy *V*_Z_ and the induced potential energy 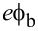. The magnetic field lowers the band bottom, charging the wire, whereas the induced potential energy, coming from electrostatic repulsion, tends to compensate that trend. In the finite-length wire, the evolution of the induced potential profile along the nanowire length (*x*-axis) for different Zeeman fields is shown in Figure 2b. As can be observed, the induced potential tends to expel charge from the center of the wire, where it is positive, while it bends downwards at its ends. On the other hand, the evolution of the potential with Zeeman field exhibits a step-like behavior with regions where it increases linearly with *V*_Z_ (red curves), screening the magnetic field effects, and regions where it remains almost constant as *V*_Z_ increases (grey curves). This causes the electron fluid to behave in an incompressible or compressible manner, respectively. This different behavior can be clearly seen in Figure 2c where the electrochemical potential at the center of the wire, given by *V*_Z_ + μ − 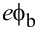
(*L*/2), is plotted as a function of the Zeeman splitting, both in the presence and absence of interactions.

**Figure 3:**
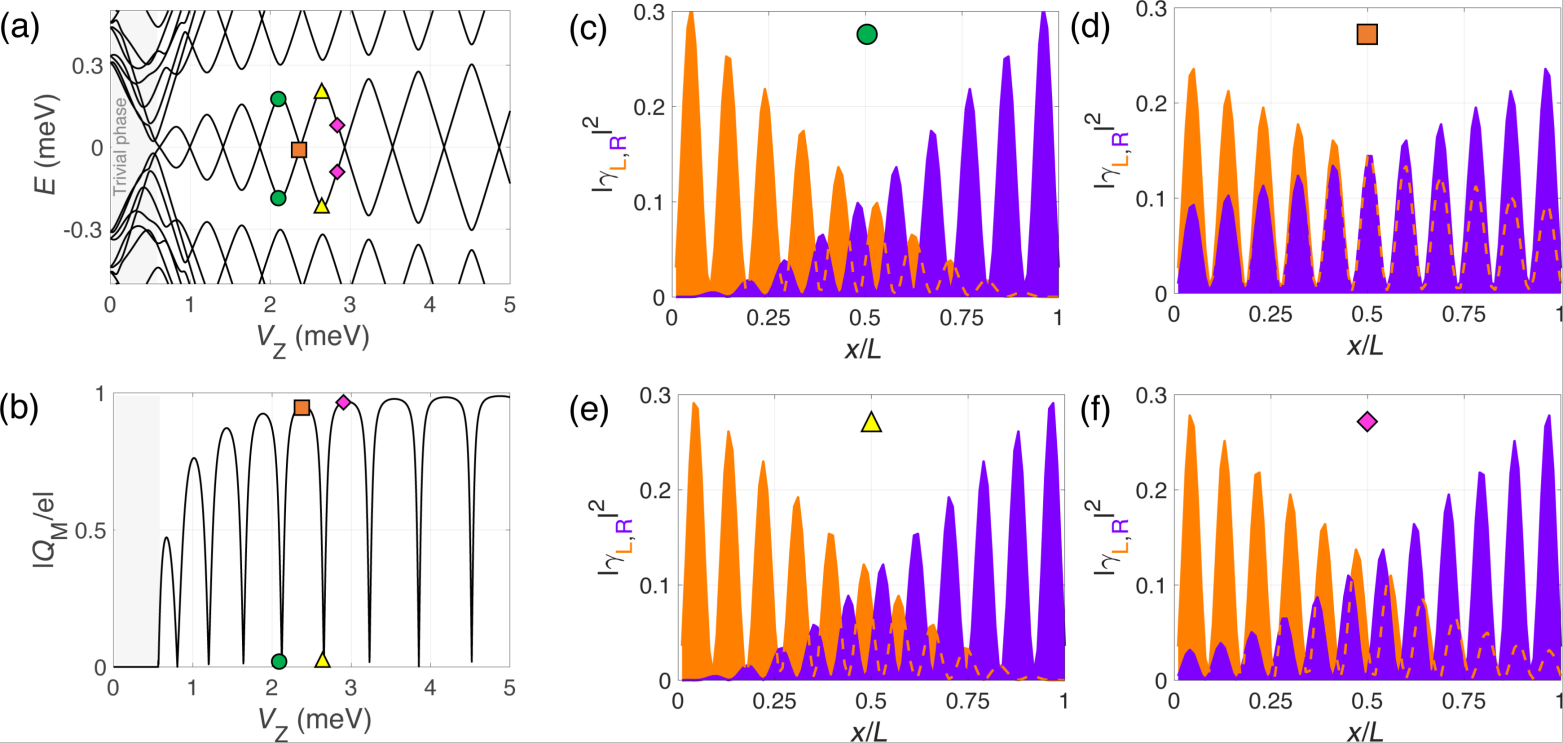
Majorana wave functions in the non-interacting case: Energy levels (a) and the absolute value of the Majorana charge *Q*_M_ (b) as functions of the Zeeman energy. Panels (c–f) show the wave-function probability profiles of the two lowest-energy states in the Majorana basis at selected values of the Zeeman field within the topological region. When the splitting is maximum (green circles and yellow triangles) the left and right Majorana wave-function oscillations are out of phase, whereas when the splitting is zero (orange square) they are in phase.

**Figure 4:**
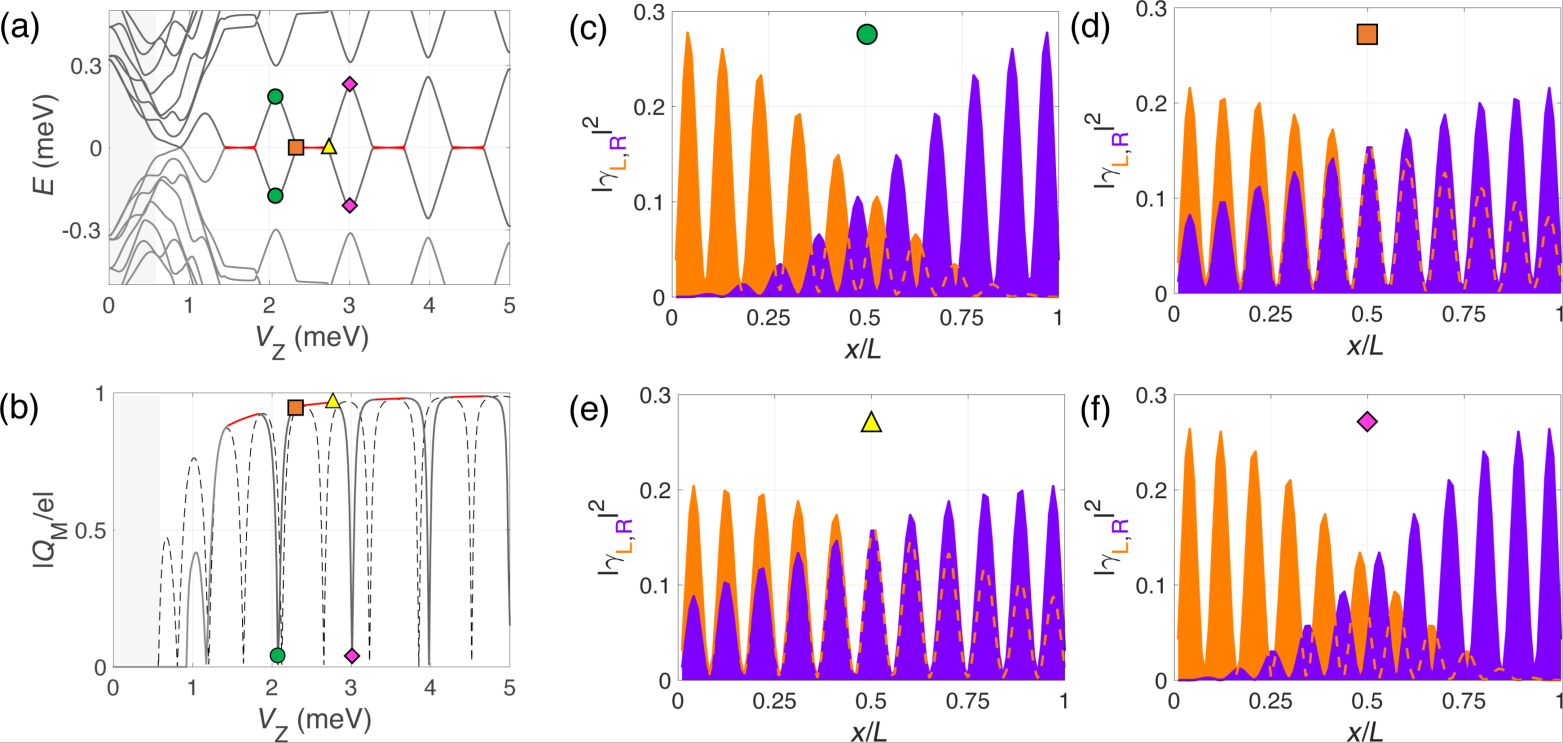
Same as Figure 3 but for the interacting case (without leads). In the pinned regions the Majorana wave functions remain in-phase as a function of the Zeeman field and the Majorana charge (b) freezes at its local maximum value (in red), instead of continuing the oscillation as in the non-interacting case (dashed curve).

The effect of this peculiar evolution of the electrostatic potential has direct consequences on the spectral properties of the wire, as we analyze below in Figure 4, but for comparison, let us first see what happens in the non-interacting case. The spectrum of the wire is shown in Figure 3a. There we can observe the emergence of low-energy subgap states for 
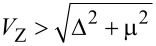
, corresponding roughly to the critical field for the bulk topological transition. We also obtain the typical energy oscillations produced by overlapping Majorana wave functions due to the finite length of the wire [21–23]. More insight can be obtained by analyzing the evolution of the total charge of the wire 
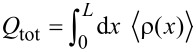
 as well as the Majorana charge *Q*_M_, the absolute value of which is given by

[3]
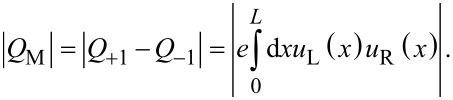


Here, *Q*_±1_ are the charges corresponding to the even/odd lowest-energy eigenstates ψ_±1_, and *u*_L,R_ are the electron components of the Majorana wave functions γ_L_ = ψ_+1_ + ψ_−1_ and γ_R_ = −i(ψ_+1_ − ψ_−1_). The total charge increases in general with magnetic field but, for finite length wires, it does so by jumping abruptly a quantity equal or smaller than *e* at each parity crossing (where the Majorana oscillations cross zero energy and the electron parity of the wire changes from even to odd or vice versa), as shown in Figure 2d, dashed curve. This abrupt change in charge is actually injected into the fermion state created by the two overlapping Majoranas and is given by |*Q*_M_| at the parity crossings. The (oscillatory) evolution of |*Q*_M_| with the magnetic field is given in Figure 3b. Strikingly, |*Q*_M_| is maximum at the parity crossing, where the energy is zero, and goes to zero at the oscillation cusps. As the length of the wire approaches to infinity, *Q*_M_ approaches zero (not shown). Indeed, the finite value of *Q*_M_ at the parity crossings is a direct measurement of the Majorana overlap, as shown in [33]. Note that the Majorana overlap is defined similarly to the right-hand side of Equation 3, but with the absolute value inside the integral.

The behavior of the Majorana wave functions is illustrated in Figure 3c–f. The probability density for the left and right Majorana wave functions exhibits an overall decay towards the center of the wire controlled by the length 
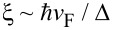
 and an oscillatory pattern controlled by λ_F_[41–42]. Moreover, the number of oscillations that fit in *L* increases by one with Zeeman field at each parity crossing. Interestingly, we observe that the left–right oscillatory patterns are out of phase for the cases where the splitting of the MBSs is maximum (Figure 3c,e. This minimizes the left–right wave function overlap and the Majorana charge goes to zero. On the other hand, the oscillations are in phase (Figure 3d) when the energy splitting is zero, at the parity crossings, producing a maximum in |*Q*_M_| and overlap. Although the Majorana wave functions are more strongly located at the wire edges, we note that the charge density of this fermionic state is uniform across the wire [34] and, thus, it is uniformly affected by the interaction with the environment when this is present.

When interactions with the image charges occur, the single-point parity crossings as a function of *V*_Z_ in the spectrum are replaced by extended regions where the subgap states remain pinned at zero energy, indicated by the red lines in Figure 4a. The abrupt jumps in *Q*_tot_ in the non-interacting case are replaced by a linear increase with increasing values of *V*_Z_ at which zero-energy pinning occurs, see Figure 2d. This is a consequence of the repulsive environment that inhibits the entrance of charge in the wire where the electron liquid behaves in an incompressible manner. On the other hand, the Majorana charge remains basically constant at the pinning plateaus, as shown in Figure 4b. The finite value of *Q*_M_ in these regions indicates that zero-energy does not imply absence of overlap between the left and right Majorana states. This is actually a common misconception that we would like to point out here. The Majorana overlap, which is a measurement of the degree of non-locality of the two Majorana wave functions, mostly depends on the length of the nanowire (and to a lesser extent on other parameters, such as the induced superconductor gap and the Rashba coupling), but it is not necessarily correlated to the Majorana energy splitting. Different mechanisms can reduce this splitting, such as interactions with the environment as studied here, smooth potential or gap profiles [21,26–28,30], or orbital magnetic effects [31], and still leave the Majorana overlap unaffected. The behavior of the Majorana wave functions in this case is illustrated in Figure 4c–f. In the pinning regions the Majorana wave functions remain practically frozen and in phase. This in turn explains why |*Q*_M_| is maximum in these regions.

The generality of these results is analyzed in Section 4 of Supporting Information File 1. There we show how the width of the pinning plateau evolves with *V*_Z_ when we change the chemical potential, the dielectric permittivity or the width of the SC shell, and the aspect ratio of the nanowire section. We find that pinning remains for any chemical potential, while it vanishes when the attractive contribution of the SC shell becomes dominant over the dielectric repulsion.

### Effect of bulk normal leads

**Figure 5:**
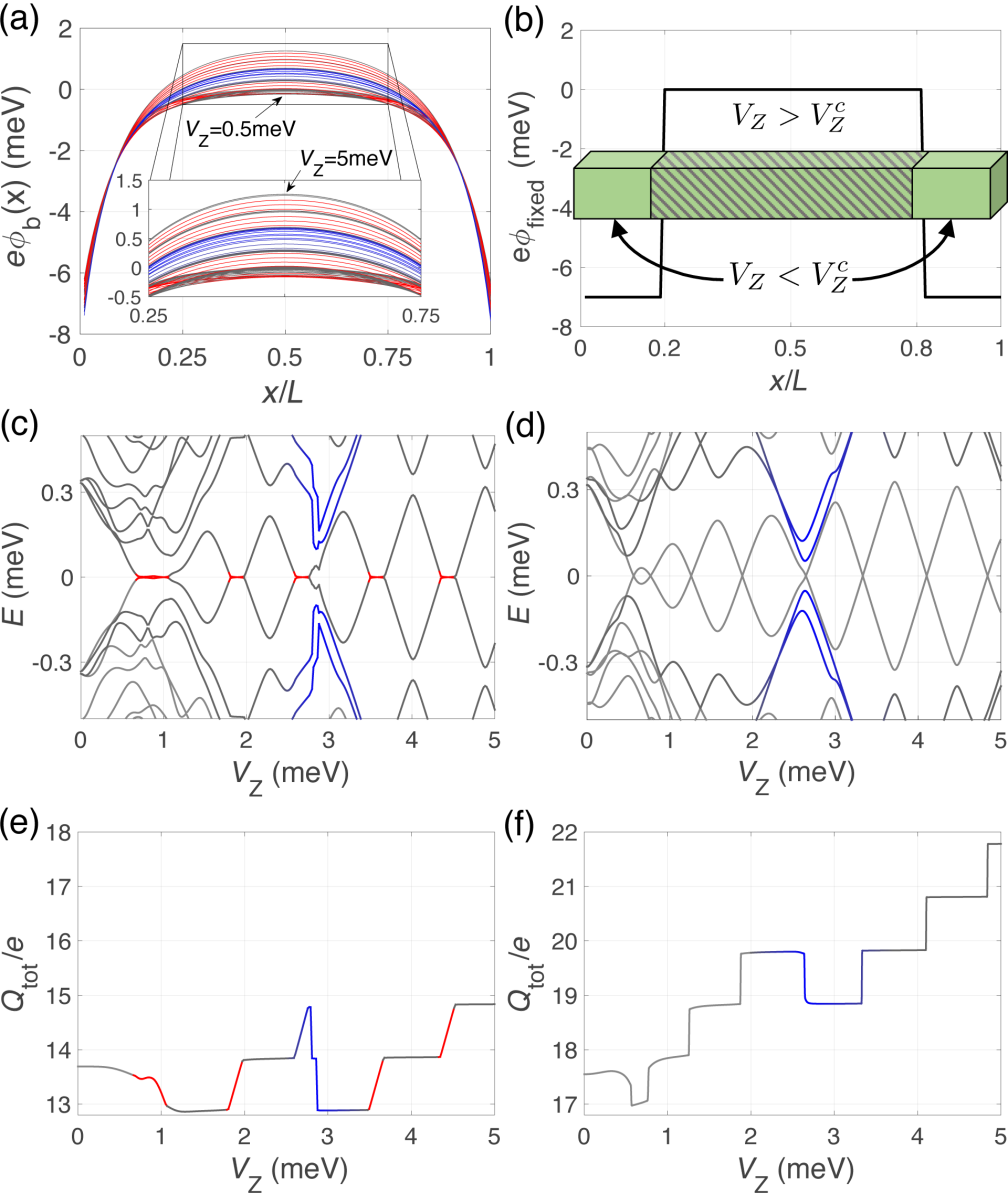
Majorana nanowire subject to interactions from the electrostatic environment (including the influence of the bulk normal leads at its ends). (a) Self-consistent induced potential energy 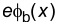 along the length of the wire for increasing values of the Zeeman splitting. The same wire parameters as in Figure 2 were used. Note that the main effect of the bulk normal leads is to create confining potential wells at the wire edges. (b) Barrier-like potential energy profile used to mimic the self-consistent solution. Spectra of the Majorana nanowire as a function of *V*_Z_ in the (c) interacting case and in the (d) non-interacting case but using the potential profile model of (b). (e, f) Evolution of the total charge *Q*_tot_ with Zeeman splitting for the two previous cases, respectively. Red color indicates incompressible electron fluid behavior as before, while blue color indicates QD-like behavior due to the metallic contacts.

In this section we analyze the effect of including the bulk normal leads in the calculation of the induced potential 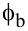. Figure 5a illustrates the evolution of 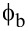 with increasing Zeeman field for the same set of parameters as in the previous section but including the normal contacts. While in the central region of the wire a similar repulsive step-like evolution with *V*_Z_ is found (corresponding to compressible/incompressible electron fluid behavior), significant attractive regions appear at the wire ends produced by the metallic character (ε_M_→∞) of the adjacent leads. As we discuss below, these attractive regions give rise to the formation of quantum-dot (QD)-like bound states that may interact with the low energy subgap states of the Majorana wire.

The evolution of the spectral properties and of the total charge *Q*_tot_ in this case are shown in Figure 5c and Figure 5e. On one hand, we observe that the pinning plateaus around each parity crossing (in red) are still present although with a smaller width. On the other hand, the main effect of the presence of the attractive potential regions is the appearance of four additional energy levels (two per contact, in blue) that approach zero energy for a value of *V*_Z_ of about 2.5–3.0 meV. At the same time we observe a rather abrupt decrease in the total wire charge (of roughly 2*e*), see Figure 5e. We can associate these additional levels with QD-like bound states arising in the attractive regions of the induced potential that anticross with the Majorana levels when their energies are on resonance [35–36,43–44].

To demonstrate the validity of this interpretation we show in Figure 5d and Figure 5f, respectively, the spectral properties and the total charge evolution for an isolated wire with a simple double potential well taken to mimic the effect of the electrostatic environment, shown in Figure 5b. Notice that in this case we do not attempt a self-consistent calculation but rather include the Zeeman field as a rigid shift of the two spin bands (like in the non-interacting case but with an inhomogeneous potential profile). Although the zero-energy pinning is not captured by this model, one can clearly observe the presence of four levels coming down towards zero energy for a value of *V*_Z_ of about 2.5–3.0 meV, as in the interacting case. The presence of these states is a consequence of the renormalization of the topological phase transition due to the electrostatic potential (either 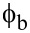 or 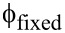):

[4]
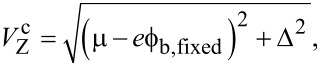


which is not constant along the wire because 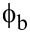 (or 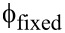) depend on *x*. For the shown values of *V*_Z_, only the central part of the wire is in the topological regime (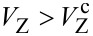), corresponding to an effectively shorter Majorana wire, whereas the outer parts are trivial (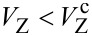), corresponding to two effective QDs attached to it. Specific details of how QD levels interact with Majorana nanowire ones can be found in [35–36,43–45].

**Figure 6:**
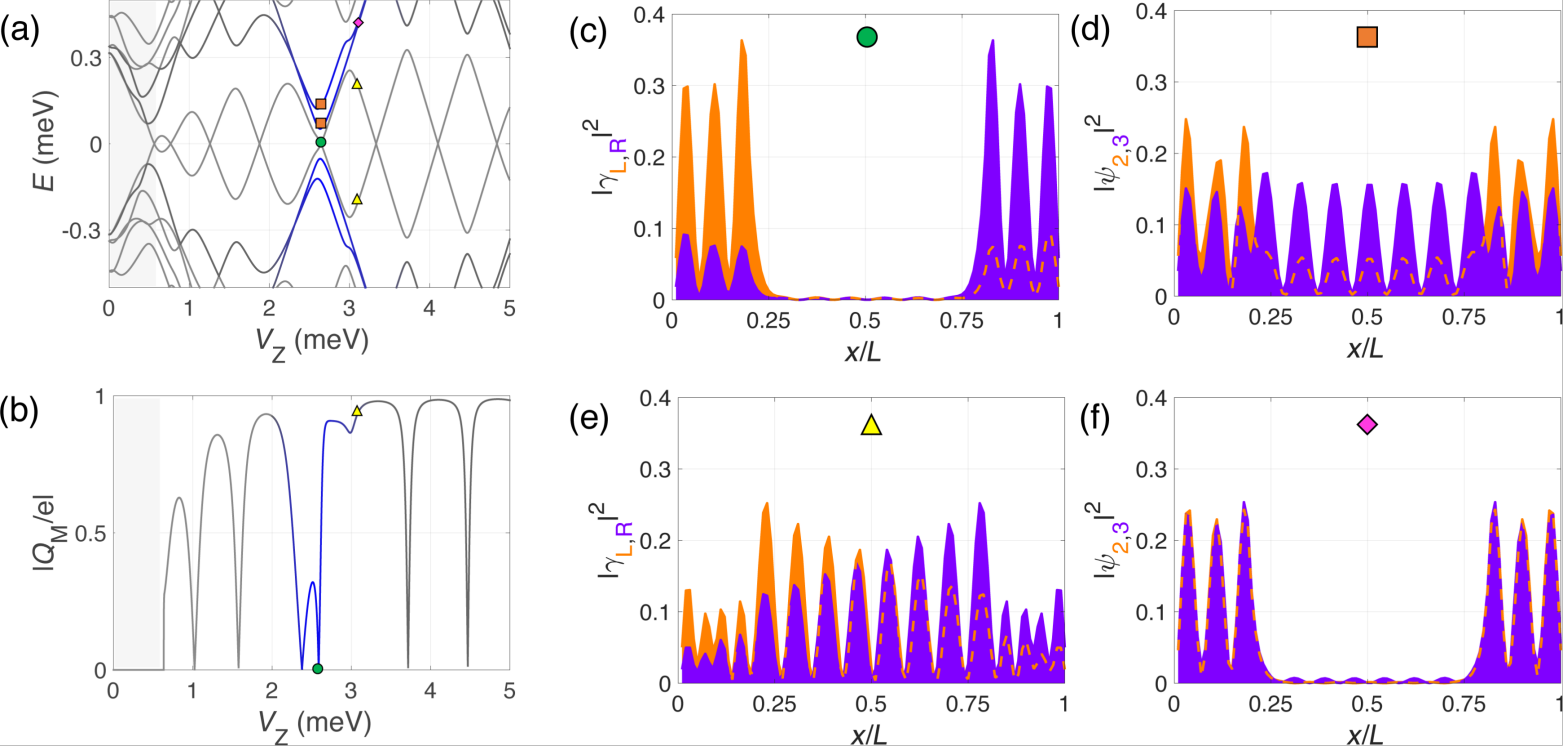
Evolution with Zeeman field of the spectrum (a) and the absolute value of the Majorana charge *Q*_M_ (b) for the barrier-like potential model of Figure 5b. Panels (c) and (e) show the wave-function probability profile (in the Majorana basis) of the two lowest-energy states at the *V*_Z_ values indicated in (a). Panels (d) and (f) show the same but for the second and third energy states (QD-like states). At the QD–Majorana levels anticrossing, the Majorana wave function leaks into the dot regions leaving the topological region of the wire practically void. This is manifested in |*Q*_M_| by two consecutive zeros, one per dot level (around *V*_Z_ = 2.5 meV).

Further information about the nature of the low-energy states at *V*_Z_≈ 3 meV is provided in Figure 6 where we plot the wave-function probability profiles (in the Majorana basis) of the low-energy states around the QD–Majorana levels anticrossing. For simplicity, we consider only the case of the potential barrier model. At the anticrossing, the Majorana and dot states merge and cannot be really told apart, but we will refer to the two lowest in energy as Majorana levels and to the other two as dot levels. As can be observed, at the anticrossing the Majorana levels (green circle) leak into the QD regions leaving the central (topological) part practically void. Conversely, the two dot-like states (immediately above in energy, orange squares) penetrate and delocalize along the wire. When the Zeeman field increases and the QD and Majorana levels are detuned, the dot states depart from low energy (pink rhombus) and from the topological part of the wire, whereas the usual overlapping behavior of the MBSs is recovered but with the Majoranas bound to the effective topological edges (yellow triangle). The absolute value of the Majorana charge as a function of *V*_Z_ is shown in Figure 6b, calculated considering only the two lowest-energy states (as before). At the anticrossing the Majorana charge oscillation is distorted, see blue region, but the area below the curve is conserved. The missing charge in Figure 5b does not come from the Majorana states, but from the dot states. At the anticrossing region, the two QD states (one per potential well) that were occupied (below the Fermi level) move upwards in energy as the Zeeman field increases and cross the Fermi level, emptying themselves. This is why in the blue regions of Figure 5e,f the total charge of the wire does not increase at the corresponding parity crossing, but instead decreases loosing effectively twice the charge of an electron *e*.

Finally, we would like to point out that, when the dot levels anticross the Majorana levels in a pinning region, the Majorana states detach from zero energy. This can be seen in Figure 5c and Figure 1b. The reason is that, although in the pinning regions the Majorana energy is zero, their wave function overlap is not. It is actually maximum, as explained when discussing Figure 4. Each QD acts as a local probe (one couples to the left topological region of the wire, the other to the right). If the length of teh wire were large (much bigger than the coherence length), left and right Majoranas would be disconnected from each other, and a local probe coupled to one of them would not be able to change its energy or perturb it. This is actually the core manifestation of their topological protection. However, when the length of the wire is finite and the Majoranas overlap, each QD couples to both Majoranas at either end and their energies are modified. The typical shapes of the anticrossing were recently analyzed and can be used to quantify the degree of Majorana non-locality [35–36].

## Conclusion

In this work we have studied the low-energy characteristics of Majorana nanowires while including their interaction with a realistic 3D electrostatic environment. This is done by solving self-consistently the Bogoliubov–de Gennes equation together with the Poisson’s equation. Typically, the total charge of the wire in equilibrium with the reservoirs increases with magnetic field (or the chemical potential of the wire). However, if the electrostatic screening is smaller inside the wire than at the contacts, a repulsive interaction arises that leads to zero-energy pinning around parity crossings in the spectrum of the wire. While the screening due to the parent SC shell tends, in general, to reduce this pinning effect, we find that it still persists depending on the quality of the SC layer and the location of the charge density within the nanowire. The pinning mechanism could help explain the precise shape of the Majorana oscillations (or lack thereof) observed in some d*I*/d*V* experiments, which exhibit substantial deviations from the predictions of simple models for finite length wires.

On the other hand, and more importantly, the self-consistent solution of the electrostatic potential varies nonhomogeneously along the wire. It is relatively flat in the central region but, due to the screening from the left/right metallic contacts, it becomes strongly negative at the edges. This creates potential wells that confine QD-like states at the ends of the wire, which appear in the spectrum as discrete states within the induced gap that disperse with Zeeman energy or chemical potential. These QD levels interact with the Majorana states in a specific way which is strongly dependent on the Majorana wave function, and particularly on its degree of spatial non-locality. The pinning mechanism and the coupling to QD-like states compete against each other, so that the pinned zero-energy plateaus may become lifted at resonance with the dot states, thus revealing their electrostatic origin (as opposed to true wave function non-locality).

## Supporting Information

File 1 Calculational details.
