# Supplementary material for: Interaction-induced zero-energy pinning and quantum dot formation in Majorana nanowires
Source: Beilstein J Nanotechnol. 2018 Aug 15;9:2171–80. doi: 10.3762/bjnano.9.203 (PMC6122061; doi:10.3762/bjnano.9.203)
Supplement: File 1 — Calculational details. [file Beilstein_J_Nanotechnol-09-2171-s001.pdf]

Supporting Information

for

Interaction-induced zero-energy pinning and quantum dot formation in Majorana nanowires

Samuel D. Escribano[1], Alfredo Levy Yeyati[2] and Elsa Prada*[1]

Address: [1]Departamento de Física de la Materia Condensada C3, Universidad Autónoma de Madrid, E-28049 Madrid, Spain and [2]Departamento de Física Teórica de la Materia Condensada C5, Condensed Matter Physics Center (IFIMAC) and Instituto Nicolás Cabrera, Universidad Autónoma de Madrid, E-28049 Madrid, Spain

Email: Elsa Prada - elsa.prada@uam.es

* Corresponding author

Calculational details

1 Expressions for the induced potential using the image-charge method

1.1 Introduction

The electrostatic potential ϕ_b produced by the environment is calculated from the interaction between the nanowire charge density and bound charges it creates at the surrounding medium shown in Figure 1 of the main text. To do this we use the method of the image charges. We model the nanowire as a semiconductor rod of square section of relative permittivity ε, length L and rectangular section of width $W = 2R$, R being the half-width. We assume that the charge density $\rho(\vec{r})$ of the nanowire is located along its symmetry axis (x-axis) as a linear charge density. The nanowire faces are in contact with different dielectric materials of permittivities ε_1 (substrate), ε_2 (superconducting shell), ε_3 and ε_4 (surrounding medium); while two metal leads of permittivities ε_{M_1} and ε_{M_2} are placed at both ends of the nanowire. In order to gain more insight into the solution of this problem, we solve first some simpler cases. In the first section we obtain the electrostatic potential created by a linear charge density placed before one, between two, and before two infinite planes. These problems can be understood as 1D problems. In the second section we obtain the potential with all four surrounding media but without the bulk leads, which can be treated as a 2D problem. Finally, in the third section we obtain the full model including also the interaction with the leads.

1.2 One dimension

1.2.1 One infinite plane

The solution of this problem can be found in textbooks on electromagnetism [1]. When one charge q is placed (at the origin of coordinates) in a medium of dielectric constant ε and at a distance R from the interface between this and another medium of permittivity ε_1, a bound charge of magnitude $\kappa_1 q$ appears spread at the interface, where

$$\kappa_1 \equiv \frac{\varepsilon - \varepsilon_1}{\varepsilon + \varepsilon_1}. \tag{S1}$$

The effect of this bound charge is equivalent to that of a point charge of the same magnitude and located at a specular distance with respect to the plane from the original charge, see Figure S1a. This is why it is called an image charge. The classical electrostatic potential due to the interaction between the original and the image charge through Coulomb's law takes the form

$$\phi_{\mathrm{b}}(x) = \frac{1}{4\pi\varepsilon\varepsilon_0} \frac{\kappa_1 q}{\sqrt{(2R)^2 + x^2}}, \tag{S2}$$

where ε_0 is the vacuum permittivity. Because ϕ_{b} is linear in q, this result can be generalized to an arbitrary 1D density charge $\rho(x)$:

$$\phi_{\mathrm{b}}(x) = \frac{1}{4\pi\varepsilon\varepsilon_0} \int \frac{\kappa_1 \rho(x')}{\sqrt{(2R)^2 + (x-x')^2}} dx'. \tag{S3}$$

As argued in [2], since the bound charges are distinguishable from the nanowire charges (cannot tunnel in and out), this potential can be directly transformed into a quantum operator as a Hartree interaction without any Fock correction. Assuming a purely local polarizability (Thomas–Fermi limit), the density operator may be transformed as $\rho(x') \to \langle \rho(x') \rangle$, which is perfectly equivalent to the above classical equation. Then, the potential takes the form

$$\phi_{\mathrm{b}}(x) = \int V_b(x,x') \langle \hat{\rho}(x') \rangle dx', \tag{S4}$$

where the kernel $V_{\mathrm{b}}(x,x') \equiv \kappa_1 / 4\pi\varepsilon\varepsilon_0 \sqrt{(2R)^2 + (x-x')^2}$ encodes the geometrical information of the interaction.

We note that Equation S4 is general for any geometry and charge density. For this reason, in the following sections we first obtain the kernel function V_{b} for a given geometry using a single original charge, and then we generalize our results to an arbitrary density $\rho(x)$ and to its corresponding quantum expression using Equation S4.

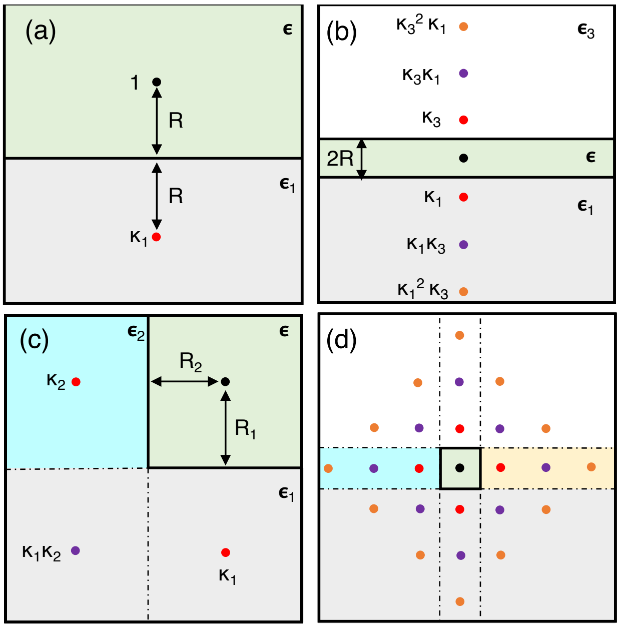

Figure S1: Configuration of the image charges produced by the original charge (in black) studied in different Sections. The original charge is in a medium of dielectric constant ε (green color in the plots) that extends in the third direction. (a) Sec. IA: a charge in front of an infinite semi-space of permittivity ε_1 at a distance R. (b) Sec. IB: a charge between two semi-infinite and parallel regions of permittivities ε_1 and ε_3. (c) Sec. IIA: a charge close to the intersection between two media of permittivities ε_1 and ε_2. (d) Sec. IIB: a charge between four different media. As before, the black point depicts the real charge, while the red, purple and orange points depict the first, second and third image charges found in the first, second and third steps of the image method procedure, respectively. For (a) and (c) all the image charges are shown, whereas for (b) and (d) there is an infinite number of them and only the first ones are shown. Black solid lines are interfaces, and black dashed lines are image interfaces.

1.2.2 Two opposite infinite planes

We now consider a charge between two infinite and parallel planes separating the dielectric constant of the nanowire ε from two other media of permittivities ε_1 and ε_3 (see Figure S1b). In order to try to satisfy the boundary conditions imposed by the Gauss' law, in a first step two image charges $\kappa_1 q$ and $\kappa_3 q$ are required in each dielectric medium at the same distance R from the interface (see red dots in Figure S1b). However, each image charge does not satisfy the boundary condition with respect to the opposite interface. For this reason, in a second step, two (accidentally equals) additional image charges of magnitude $\kappa_1\kappa_3 q$ are required at a distance $3R$ from each interface (see purple dots in Figure S1b). But, again, these additional image charges do not satisfy the boundary conditions with

respect to the opposite interfaces and another step must be taken. It is possible to see that for each image charge $q_{\alpha}^{(n)}$ in the dielectric α created at the n-th step of the image charge method, another image charge

$$q_{\beta}^{(n+1)} = \kappa_{\beta} q_{\alpha}^{(n)} \tag{S5}$$

is required in the opposite β dielectric at a distance $2\,(n+1)\,R$ from the original charge q in order to satisfy the boundary conditions, with $q_{\alpha,\beta}^{(0)} = 1$. Thus, the interaction kernel takes the form of an infinite series

$$V_{\mathrm{b}}\,(x) = \frac{1}{4\pi\varepsilon\varepsilon_0} \sum_{n=1}^{\infty} \left(\frac{q_1^{(n)} + q_3^{(n)}}{\sqrt{x^2 + (2nR)^2}} \right). \tag{S6}$$

Note that each term of the sum decreases with n as $V_{\mathrm{b}}^{(n)} \sim \kappa^n/n$. Since $|\kappa| < 1$ for a dielectric medium and $\kappa_{\mathrm{M}} = -1$ for a metallic one (where $\varepsilon_{\mathrm{M}} \to \infty$), then the kernel converges to $V_{\mathrm{b}} \sim \ln(1-\kappa)$ in spite of the infinite summation.

1.2.3 Two consecutive infinite planes

Finally, we consider the case depicted in Figure S2a where a (real) charge q is placed in a dielectric material characterized by a permittivity ε_{A}. This charge is at a distance R from a first interface with a material of dielectric constant ε_{B} and width W, and at a distance $R+W$ from a second (parallel) interface with another medium of permittivity ε_{C}. As we know, the real charge q creates an image charge $\kappa_{\mathrm{B}} q$ at a distance $2R$ from it inside the ε_{B} medium. However, the potential created by both charges is only valid inside the ε_{A} medium. The potential created inside the ε_{B} medium can be found using the image method [1]: It is the same potential than that created by an image charge of magnitude $q[2\varepsilon_{\mathrm{A}}/(\varepsilon_{\mathrm{A}}+\varepsilon_{\mathrm{B}})]$ located at the same position of the real charge. From this point, the same steps explained in the previous subsection can be followed, and once the infinite series is obtained, the potential can be transformed back to the ε_{A} medium. Thus, one can prove that the

bound charges potential is given by

$$V_{\rm b} = \frac{q}{4\pi\varepsilon_{\rm A}\varepsilon_0}\left(\frac{1}{\varepsilon_{\rm A}+\varepsilon_{\rm B}}\right)\left[\frac{\varepsilon_{\rm A}-\varepsilon_{\rm B}}{\sqrt{x^2+(2R)^2}}+ \right.$$
$$\left. + \frac{4\varepsilon_{\rm A}\varepsilon_{\rm B}}{\varepsilon_{\rm B}-\varepsilon_{\rm A}}\sum_{n=1}^{\infty}\frac{\left(\frac{(\varepsilon_{\rm B}-\varepsilon_{\rm A})(\varepsilon_{\rm B}-\varepsilon_{\rm C})}{(\varepsilon_{\rm B}+\varepsilon_{\rm A})(\varepsilon_{\rm B}+\varepsilon_{\rm C})}\right)^n}{\sqrt{x^2+(2R+2nW)^2}}\right]. \quad (S7)$$

Since this expression is rather complex, it is convenient to replace the effect of both media $\varepsilon_{\rm B}$ and $\varepsilon_{\rm C}$ by just one characterized by an effective permittivity $\varepsilon_{\rm eff}$ which, from an electrostatic point of view, is equivalent. Hence, the bound charges potential would be given by Equation S2 with $\varepsilon \to \varepsilon_{\rm A}$ and $\varepsilon_1 \to \varepsilon_{\rm eff}$. Comparing both equations, the effective permittivity (at $x = 0$) is

$$\varepsilon_{\rm eff} = \varepsilon_{\rm A}\frac{1-\kappa_{\rm eff}}{1+\kappa_{\rm eff}}, \quad (S8)$$

where

$$\kappa_{\rm eff} = \frac{1}{\varepsilon_{\rm A}+\varepsilon_{\rm B}}\left[\varepsilon_{\rm A}-\varepsilon_{\rm B}+ \right.$$
$$\left. +\frac{4\varepsilon_{\rm A}\varepsilon_{\rm B}}{\varepsilon_{\rm B}-\varepsilon_{\rm A}}\sum_{n=1}^{\infty}\frac{1}{1+n\frac{W}{R}}\left(\frac{(\varepsilon_{\rm B}-\varepsilon_{\rm A})(\varepsilon_{\rm B}-\varepsilon_{\rm A})}{(\varepsilon_{\rm B}+\varepsilon_{\rm C})(\varepsilon_{\rm B}+\varepsilon_{\rm C})}\right)^n\right]. \quad (S9)$$

We note that this system corresponds to the nanowire–SC shell–vacuum double interface of Figure 1a of the main text. There, the effect of the shell finite width and the vacuum on top has been condensed in an effective SC permittivity. This means that, in Equation S8, $\varepsilon_{\rm eff} \to \varepsilon_{\rm SC}$, $\varepsilon_{\rm A} \to \varepsilon$, $\varepsilon_{\rm C} \to \varepsilon_{\rm a}$ and $\varepsilon_{\rm B}$ is the true SC thin film permittivity $\varepsilon_{\rm tf}$.

In Figure S2b we show the effective SC permittivity $\varepsilon_{\rm SC}$ considered in the main text as a function of $\varepsilon_{\rm tf}$ for two different shell widths $W_{\rm SC}$. Notice that, as the SC shell becomes thinner (red curve corresponds to 8 nm), the effective permittivity gets more renormalized. A value $\varepsilon_{\rm SC} \sim 100$ corresponds to $\varepsilon_{\rm tf} \sim 4000$.

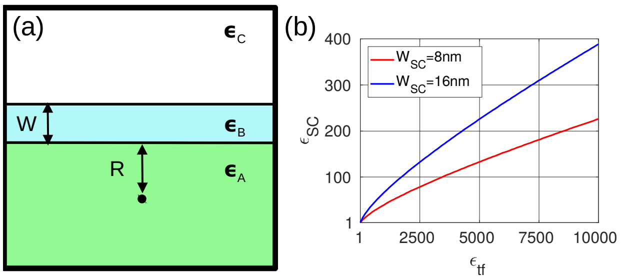

Figure S2: (a) System studied in Sec. IC. A charge (black dot) is in a medium of dielectric constant ε_A (the nanowire) and placed at a distance R from a thin material of permittivity ε_B and thickness W (the SC shell). Above the thin film there is another semi-infinite space of permittivity ε_C (the vacuum). When all the permittivities are finite, bound charges arise in both surrounding mediums. (b) Effective SC permittivity ε_{SC} calculated using Equaiton S8 vs the SC thin film permittivity ε_{tf} for two different film thicknesses W_{SC}. Parameters are the same as in the main text.

1.3 Two dimensions

1.3.1 One rectangular corner

This is also a textbook problem [1]. A charge q inside a dielectric with permittivity ε is placed at a distance R_1 from dielectric ε_1 and at a different distance R_2 form dielectric ε_2, which are perpendicular to one another (see Figure S1c). To satisfy the boundary conditions, two image charges $\kappa_1 q$ and $\kappa_2 q$ are required in each dielectric at $(x,-2R_1,0)$ and $(x,0,-2R_2)$, see red dots. Because of these, another image charge of magnitude $\kappa_1\kappa_2 q$ is required at $(x,-2R_1,-2R_2)$, purple dot. In this case, and due to the closed geometry of the problem, three image charges are enough to satisfy the boundary conditions. The kernel function takes thus the form

$$V_b(x) = \frac{1}{4\pi\varepsilon\varepsilon_0}\left(\frac{\kappa_1}{\sqrt{x^2+(2R_1)^2}} + \frac{\kappa_2}{\sqrt{x^2+(2R_2)^2}} + \right.$$
$$\left. + \frac{\kappa_1\kappa_2}{\sqrt{x^2+(2R_1)^2+(2R_2)^2}}\right). \qquad \text{(S10)}$$

1.3.2 Four rectangular corners: interaction without the leads

Now a charge q is placed inside an infinite wire of rectangular section and of permittivity ε, see Figure S1d. This charge is at a distance R_1 from two parallel flat dielectrics with permittivities ε_1

and ε_3, and at a distance R_2 from another two parallel dielectrics with permittivities ε_2 and ε_4 which are perpendicular to the previous ones. Combining what we have learned in the previous Sections, to satisfy the boundary conditions an infinite ensemble of image charges have to be placed in the different dielectrics as shown in Figure S1d. For each image charge in one dielectric, another one appears at a specular distance from the opposite interface, while for each two image charges placed in two perpendicular media, just one more appears at the corner. The electrostatic potential due to all these charge is given by

$$V_{\mathrm{b}}(x) = \frac{1}{4\pi\varepsilon\varepsilon_0}\left[\sum_{n,m=1}^{\infty}\left(\frac{\left(q_1^{(n)}+q_3^{(n)}\right)\left(q_2^{(m)}+q_4^{(m)}\right)}{\sqrt{x^2+(2nR_1)^2+(2mR_2)^2}}\right)+ \right.$$
$$\left. +\sum_{n=1}^{\infty}\left(\frac{q_1^{(n)}+q_3^{(n)}}{\sqrt{x^2+(2nR_1)^2}}+\frac{q_2^{(n)}+q_4^{(n)}}{\sqrt{x^2+(2nR_2)^2}}\right)\right], \tag{S11}$$

where:

$$\begin{cases} q_1^{(n+1)} = \kappa_1 q_3^{(n)}, \qquad q_2^{(n+1)} = \kappa_2 q_4^{(n)}, \\ q_3^{(n+1)} = \kappa_3 q_1^{(n)}, \qquad q_4^{(n+1)} = \kappa_4 q_2^{(n)}, \\ q_\alpha^{(0)} = 1 \ \forall \alpha = \{1,2,3,4\}. \end{cases} \tag{S12}$$

If the wire's section is square ($R_1 = R_2$), then the kernel function can be rewritten in a more compact way:

$$V_{\mathrm{b}}(x) = \frac{1}{4\pi\varepsilon\varepsilon_0}\sum_{n,m=0}^{\infty}\left(\frac{\left(q_1^{(n)}+q_3^{(n)}-\delta_{n,0}\right)\left(q_2^{(m)}+q_4^{(m)}-\delta_{m,0}\right)}{\sqrt{x^2+(2nR)^2+(2mR)^2}}\right)(1-\delta_{n+m,0}). \tag{S13}$$

Note that the number of charges increases as $4n$ at each n-th step of the image charge method, whereas the rest of the expression inside the brackets decreases as κ^n/n as before. Thus, each term

of the sum goes as $V_\mathrm{b}^{(n)} \sim \kappa^n$ and the infinite sum is proportional to $V_\mathrm{b} \sim \kappa/(1-\kappa)$ if $|\kappa| < 1$, so the convergence of the kernel is ensured in this case as well.

1.4 The full model

Finally, we solve the full system of Figure 1 of the main text. Now, apart from the four dielectric media in each of the four faces of the square section, there are another two faces in the x-direction in contact to metallic regions. We consider that the nanowire has a square section of semi-width R. First, we assume that the charge q is placed at the coordinates origin and at the same distance R from each metallic region M_1 and M_2. Following the same procedure as before we obtain

$$V_\mathrm{b}(x) = \frac{1}{4\pi\varepsilon\varepsilon_0} \sum_{n,m,k=0}^{\infty} \left(\frac{\left(q_1^{(n)} + q_3^{(n)} - \delta_{n,0}\right)\left(q_2^{(m)} + q_4^{(m)} - \delta_{m,0}\right) q_{M_1}^{(k)}}{\sqrt{(x+2kR)^2 + (2nR)^2 + (2mR)^2}} + \right. \\ \left. + \frac{\left(q_1^{(n)} + q_3^{(n)} - \delta_{n,0}\right)\left(q_2^{(m)} + q_4^{(m)} - \delta_{m,0}\right)\left(q_{M_2}^{(k)} - \delta_{k,0}\right)}{\sqrt{(x-2kR)^2 + (2nR)^2 + (2mR)^2}} \right) \left(1 - \delta_{n+m+k,0}\right), \quad \text{(S14)}$$

where

$$\begin{cases} q_{M_1}^{(n+1)} = \kappa_{M_1} q_{M_2}^{(n)}, \qquad q_{M_2}^{(n+1)} = \kappa_{M_2} q_{M_1}^{(n)}, \\ q_\alpha^{(0)} = 1 \;\; \forall \alpha = \{M_1, M_2\}. \end{cases} \quad \text{(S15)}$$

If now the charge q is placed at an arbitrary position x' inside the nanowire, and the metal M_1 interface is at $x = 0$ and the M_2 interface is at $x = L$, then the kernel function is given by

$$V_{\mathrm{b}}(x) = \frac{1}{4\pi\varepsilon\varepsilon_0} \sum_{n,m,k=0}^{\infty} \left(\frac{\left(q_1^{(n)} + q_3^{(n)} - \delta_{n,0}\right)\left(q_2^{(m)} + q_4^{(m)} - \delta_{m,0}\right) q_{M_1}^{(k)}}{\sqrt{\left(x - (-1)^k \left(2^{\mathrm{floor}\left(\frac{k}{2}+1\right)} L - 2L + x'\right)\right)^2 + (2nR)^2 + (2mR)^2}} + \right.$$
$$\left. + \frac{\left(q_1^{(n)} + q_3^{(n)} - \delta_{n,0}\right)\left(q_2^{(m)} + q_4^{(m)} - \delta_{m,0}\right)\left(q_{M_2}^{(k)} - \delta_{k,0}\right)}{\sqrt{\left(x + (-1)^k \left(2^{\mathrm{floor}\left(\frac{k+1}{2}\right)} L - x'\right)\right)^2 + (2nR)^2 + (2mR)^2}} \right) \left(1 - \delta_{n+m+k,0}\right). \quad \text{(S16)}$$

If L is large enough compared to $2R$, we can take into account only the lowest order of the image charges at the metals, $q_{M_i}^{(k=0,1)}$, and the number of charges at each n-th step of the image charge method increases only as $\sim 12n$. Then, the system follows the same convergence criterion as in the previous section. If $L \sim 2R$, Equation S16 converges as well, but the demonstration is longer.

2 Further details on numerical methods

2.1 Mean field approximation to treat electron-electron interactions

We want to solve the energy spectrum of the nanowire Hamiltonian $\hat{H}$ when the interaction between electrons $\hat{\phi}$ is included. In general, this interaction can be written in second quantization as

$$\hat{\phi} = \sum_{\alpha,\beta} \check{c}_\alpha^\dagger \check{c}_\alpha V_{\alpha\beta} \check{c}_\beta^\dagger \check{c}_\beta, \quad \text{(S17)}$$

where $\check{c}_\alpha^\dagger$, $\check{c}_\alpha$ are defined as the *Nambufied* vector of operators

$$\check{c}_\alpha^\dagger = \left(c_{1\uparrow}^\dagger, c_{1\downarrow}^\dagger, c_{2\uparrow}^\dagger, ..., c_{N\downarrow}^\dagger, c_{1\uparrow}, c_{1\downarrow}, c_{2\uparrow}, ..., c_{N\downarrow}\right). \quad \text{(S18)}$$

Here, $c_{i\sigma}^\dagger$ and $c_{i\sigma}$ are electron creator/annihilation operators with quantum numbers α. $V_{\alpha\beta}$ above encodes the electronic interaction. Therefore, Greek indexes α, β encode both particle/hole char-

acter, spin ($\uparrow,\downarrow$), and any other indexes the electron might have, such as site index $i = 1,...,N$ in a tight-binding description.

To treat the quartic interaction we resort to a mean field approach called the Hartree–Fock–Bogoliubov (HFB) approximation. Using the Wick's theorem and neglecting fluctuations and constant terms we can write

$$\hat{\phi}_{\text{eff}} = \sum_{\alpha,\beta} V_{\alpha\beta} \left[\left\langle \check{c}^{\dagger}_{\alpha}\check{c}_{\alpha} \right\rangle \check{c}^{\dagger}_{\beta}\check{c}_{\beta} + \left\langle \check{c}^{\dagger}_{\beta}\check{c}_{\beta} \right\rangle \check{c}^{\dagger}_{\alpha}\check{c}_{\alpha} + \left\langle \check{c}^{\dagger}_{\alpha}\check{c}_{\beta} \right\rangle \check{c}^{\dagger}_{\beta}\check{c}_{\alpha} + \right.$$

$$\left. + \left\langle \check{c}_{\alpha}\check{c}^{\dagger}_{\beta} \right\rangle \check{c}^{\dagger}_{\alpha}\check{c}_{\beta} - \left\langle \check{c}^{\dagger}_{\alpha}\check{c}^{\dagger}_{\beta} \right\rangle \check{c}_{\alpha}\check{c}_{\beta} - \left\langle \check{c}_{\alpha}\check{c}_{\beta} \right\rangle \check{c}^{\dagger}_{\alpha}\check{c}^{\dagger}_{\beta} \right]. \tag{S19}$$

The first two terms are known as Hartree terms, which include information about direct (repulsive/attractive) interaction between electrons. The second and third are the Fock terms, which include the exchange interaction due to the electron indistinguishable properties. These terms ensure that non-physical self-interactions introduced by the first terms are cancelled. The last two are known as Bogoliubov terms, which include possible pairing correlations between electrons.

We want to rewrite Equation S19 in a more compact manner. In order to do that, we define two matrices:

- The lambda matrix:

  $$\Lambda \equiv \mathbb{I}_{space} \otimes \mathbb{I}_{2\times 2} \otimes \sigma_x, \tag{S20}$$

  where $\mathbb{I}_{space}$ is the identity matrix in real space (for a one-dimensional tight-binding model with N sites, this matrix is the $N \times N$ identity), $\mathbb{I}_{2\times 2}$ is the identity matrix in spin space, and σ_x is the Pauli x-matrix in Nambu space. This matrix satisfies the property $\check{c}^{\dagger} = \Lambda\check{c}$.

- The density matrix:

$$\rho_{\alpha\beta} \equiv \left\langle \check{c}_{\alpha}^{\dagger} \check{c}_{\beta} \right\rangle. \tag{S21}$$

We can express this matrix in terms of the eigenvectors γ_n of the Hamiltonian $\hat{H} + e\hat{\phi}_{\text{eff}}$, which are related to the $\check{c}_{\alpha}$'s through a unitary transformation Ψ as $\gamma_n = \Psi_{n\alpha}\check{c}_{\alpha}$. Then

$$\rho_{\alpha\beta} = \left\langle \check{c}_{\alpha}^{\dagger} \check{c}_{\beta} \right\rangle = \Psi_{\alpha n}^{*} \left\langle \gamma_n^{\dagger} \gamma_m \right\rangle \Psi_{m\beta} = \left(\Psi^{\dagger} F \Psi \right)_{\alpha\beta}, \tag{S22}$$

where $F_{nm} \equiv \langle \gamma_n^{\dagger} \gamma_m \rangle = f_{FD}(\varepsilon_n)\, \delta_{nm}$ is the Fermi–Dirac distribution matrix.

Using these two matrices, Equation S19 can be rewritten as

$$\begin{aligned} \phi_{\text{eff}} = 2\mathscr{D}[V \cdot d\{\rho\}] + \Lambda \cdot (V \star \rho) \cdot \Lambda + V \star (\Lambda \cdot \rho \cdot \Lambda) - \\ - \Lambda \cdot (V \star (\rho \cdot \Lambda)) - (V \star (\Lambda \cdot \rho)) \cdot \Lambda, \end{aligned} \tag{S23}$$

where we assume a symmetric potential $V_{\alpha\beta} = V_{\beta\alpha}$. Here we have used the notation $\mathscr{D}[v]$ as the diagonal matrix with vector v in its diagonal, $d\{A\}$ as a column vector whose elements are the diagonal elements of matrix A, the dot product $A \cdot B$ as a matrix product, and the star product $A \star B$ as an element wise product between matrices.

However, Equation S23 does not have Nambu structure because in general $V_{i\sigma\tau,i\sigma\tau} \neq V_{i\sigma\bar{\tau},i\sigma\bar{\tau}}$, so Bogoliubov–de Gennes formalism cannot be applied. We symmetrize this expression by doing

$$\check{c}^{\dagger} \phi_{\text{eff}} \check{c} = \check{c}^{\dagger} \left(\frac{\phi_{\text{eff}} - \Lambda \phi_{\text{eff}}^{t} \Lambda}{2} \right) \check{c} + \text{cnst.}, \tag{S24}$$

where we have used the anticommutation relation $\{c, c^{\dagger}\} = 1$, the property $\Lambda^{t} = \Lambda$ and we neglect constant terms again. Thus, the general interaction between electrons in the HFB approximation can

be written as

$$\phi^{\mathrm{HFB}} = \frac{1}{2}\check{c}^{\dagger}\left(\phi_{\mathrm{eff}} - \Lambda\phi_{\mathrm{eff}}^{t}\Lambda\right)\check{c}, \tag{S25}$$

where ϕ_{eff} is given by Equation S23.

2.2 Inclusion of the intrinsic interaction

The interaction between the electrons inside the nanowire (intrinsic interaction) is given, in principle, by the bare Coulomb potential in one dimension. Taking into account the finite radius (half-width) R of the wire, a more precise form for the interaction is [3]

$$V(x) = \frac{\sqrt{\pi}}{4\pi\varepsilon\varepsilon_0 R} e^{x^2/R^2} \mathrm{Erfc}\left(\frac{|x|}{R}\right), \tag{S26}$$

where x is the distance between electrons.

When we consider this potential only at the Hartree level, we find zero energy pinning around parity crossings, just like we did with the extrinsic interaction. However, this pinning is unphysical since it comes from spurious self-interaction terms introduced by the Hartree approximation [2]. For the intrinsic interaction it is thus necessary to include the Fock correction due to the indistinguishability of electrons in the nanowire.

If we consider the bare interaction, Equation S26, in the Fock terms, an overcompensation of the pinning effect is found and unphysical jumps appear at each parity crossing. To cure this problem, we introduce screening in the quasi-static Thomas–Fermi limit so that the potential in the Fock terms acquires an additional exponential decay $e^{-|x|/\lambda_{\mathrm{TF}}}$ that depends on the Thomas–Fermi length λ_{TF}, which should be of the order of the Fermi wavelength. In this case, we find that the parity crossings induced by the intrinsic interaction are suppressed, in agreement with the self-interaction argument.

If the nanowire is discretized using a tight-binding model, the interaction can be written as

$$V_{\alpha,\beta} = \frac{\sqrt{\pi}}{4\pi\varepsilon\varepsilon_0 R}\exp\left\{\left(\frac{i-j}{aR}\right)^2 - \frac{|i-j|}{a\lambda_{\text{TF}}}\right\}\cdot$$

$$\cdot\,\text{Erfc}\left(\frac{|i-j|}{aR}\right)\left[1-\delta_{\alpha,\beta}\right], \quad \text{(S27)}$$

where the indexes $\alpha = \{i,\sigma,\tau\}$ and $\beta = \{j,\sigma',\tau'\}$ include all the quantum numbers of the electrons, a is the distance between two neighboring sites (lattice constant), and the term $\left[1-\delta_{\alpha,\beta}\right]$ ensures that an electron cannot interact with itself. As stated before, the above equation is only valid for the Fock terms, while for the Hartree terms it is the bare interaction (same expression with $\lambda_{\text{TF}} \to \infty$). Then, one can obtain the potential in the HFB approximation using Equation S25.

The electron–electron interaction between the nanowire and the bound charges of the dielectric environment (*extrinsic* interaction) is found in Section 1, and it may be implemented following the same procedure by substituting $x \to (i-j)/a$. We note that now the term 1 $\left[1-\delta_{\alpha,\beta}\right]$ should not be included since electron α is always inside the nanowire while β is outside, in the surrounding medium (or the other way around). Finally, the potential in the HFB approximation can be computed using Equation (S25), but now the Fock and the Bogoliubov terms have to be ignored as we argued in Section 1, so that the last four terms of Equation S23 are not considered.

2.3 Numerical self-consistent method used to solve the eigenspectrum

We note that $\hat{H} + e\hat{\phi}^{\text{HFB}}$ depends on its own eigenvectors (see Equation S22), and thus it has to be solved self-consistently. We solve this problem numerically using the following procedure: in the first iteration of the self-consistent method, we find the density-matrix ρ using the eigenvectors of the Hamiltonian $\hat{H}$ as a test solution. In the next step we obtain a new ρ diagonalizing $\hat{H} + e\hat{\phi}^{\text{HFB}}$ where the interaction has been obtained using the previous density matrix. In the following steps, the density matrix is found using a linear combination of the eigenvectors of $\hat{H} + e\hat{\phi}^{\text{HFB}}$ in the two previous iterations. This is done to introduce damping in the iteration procedure in order to ensure the convergence of the self-consistent method. In each step, we compare the eigen-energies of

$\hat{H} + e\hat{\phi}^{\mathrm{HFB}}$ with those of the previous step. We repeat this procedure until convergence. We consider the iteration has converged when the difference between both energy spectra is much smaller than the main energy scale of our problem i.e., the superconductor gap Δ.

3 Nanowire spectrum including the intrinsic interaction

Here we show that the features studied in the main text (zero-energy pinning and QD formation) remain qualitatively unaltered when including electron–electron interactions ϕ_{int} inside the nanowire. The intrinsic interaction introduces small quantitative changes in the spectrum, but the qualitative behavior stays the same. Following our previous work [2], we treat this interaction at the mean field level, within the Hartree–Fock–Bogoliubov approximation, and assume a bare Coulomb interaction for the Hartree terms and a screened Coulomb interaction in the quasi-static Thomas–Fermi limit for the Fock terms (see Section 2 for more details).

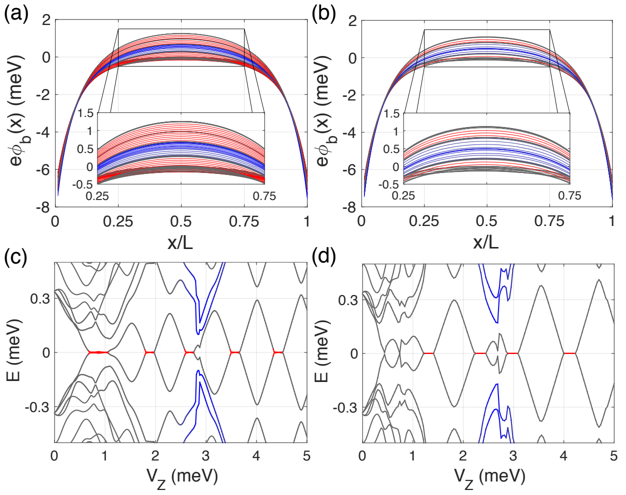

Figure S3: Majorana nanowire in the presence of interactions (including the influence of the bulk normal leads at its ends). Self-consistent induced potential energy $e\phi_b(x)$ along the length of the wire for increasing values of the Zeeman splitting ignoring (a) and including (b) the electron-electron interactions inside the nanowire. (c) and (d) are their corresponding energy spectra. Wire parameters are the same as in the main text, and the Thomas–Fermi length is $\lambda_{\mathrm{TF}} = L/3$

In Figure S3 we show the bound charges electrostatic potential along the nanowire and the energy spectrum versus the Zeeman field ignoring (Figure S3a,c) and including (Figure S3b,d) the intrinsic interaction. Note that here we do not include the Bogoliubov correction since this term just renor-

malizes the gap as shown in [2]. In general, both spectra are qualitatively similar and thus we can conclude that the intrinsic interactions do not alter the features studied in the main text. However, there are some quantitative differences. First, the dispersive QD levels approach zero energy at a slightly smaller Zeeman energy as a result of small changes in the induced potential ϕ_b, as can be seen in Figure S3d. Second, the position of the gap closing and thus, the topological phase transition, shifts to a different magnetic field. This is also a consequence of small changes in ϕ_b, as well as small changes in the Zeeman energy induced by the Fock terms, that modify the value of the critical Zeeman field through Equation 4 of the main text. Finally, the energy splitting of the Majoranas is larger due to the renormalization of the Fermi momentum induced by the Fock terms as well.

4 Robustness of the pinning effect

We want to test the validity of our results when varying different parameters of the electrostatic environment and the location of the Majorana wave function across the nanowire section. Figure S4 provides various phase diagrams indicating the occurrence of MBS zero-energy pinning (in red) as a function of the different parameters. Although we have taken $\mu = 0.5$meV for the simulations of the main text, pinning is general for all chemical potentials (within the topological phase), as can be seen in the upper panels of Figure S4. As a function of μ and V_Z, the non-interacting lines of Figure S4 a corresponding to point-like parity crossings transform into incompressible finite width stripes in the interacting case (Figure S4b). Pinning regions are bigger for lower chemical potentials and for higher magnetic fields, since the repulsive interaction is larger too. It can also be observed that the onset of the topological phase is different in the interacting system than in the non-interacting one (black dashed line), at least for positive μ. This is because the electrostatic potential renormalizes the chemical potential [4] and thus it modifies the value of the bulk critical magnetic field given in Equation 4 of the main text.

However, pinning is not general for all kind of environments or Majorana charge density position. Figure S4c shows the zero energy regions across the $V_Z - \varepsilon_{SC}$ space (the $\mu - \varepsilon_{SC}$ diagram exhibits a similar behavior). For $\varepsilon_{SC} \gtrsim 300$ the pinning plateau width shrinks into points because the elec-

trostatic environment turns into an attractive one. This means that bound charges of the opposite sign arise in the dielectric medium at these large permittivities, so that the entrance of charge at each parity crossing is no longer suppressed. Note that $\varepsilon_{\mathrm{SC}}$ represents the effective SC permittivity of the system composed by a SC thin film (epitaxially grown over the nanowire) of intrinsic permittivity $\varepsilon_{\mathrm{tf}}$, finite width W_{SC} and covered by vacuum, as we argue in Section 1.2.3. For a film width of 8 nm, an effective $\varepsilon_{\mathrm{SC}} \gtrsim 300$ corresponds to a SC permittivity of $\varepsilon_{\mathrm{tf}} \gtrsim 15000$, i.e., basically a perfect metal. The width of the nanowire also plays a role in the appearance of pinned regions. Figure S4d shows the incompressible regions as a function of V_{Z} and r_{yz}, where $r_{yz} = W_y/W_z$ is the aspect ratio of the nanowire section. When the distance between the SC shell and the opposite side is large (large r_{yz}), pinning is bigger. This is because the relative coverage of the wire by the SC shell decreases and so does its attractive contribution.

Finally, if we consider perfect metallic screening by the SC shell, i.e., $\varepsilon_{\mathrm{SC}} \to \infty$, we can also study the appearance of the pinned regions depending on the distance between the nanowire charge density and the SC shell. In Figure 4e we study the phase diagram as a function of V_{Z} and the position of the charge density across the nanowire section, y/R, where R is the wire's half width. In Figure 4f the position is fixed to the center of the wire, but we vary the aspect ratio of the wire. In both cases we observe that, as the Majorana wave functions approaches the SC, the screening effect increases and the pinning disappears [5]. However, if the wave function separates from the SC shell, the pinning survives. This may happen when the bottom gate attracts the charge density away from the SC or when the wave function is more spread throughout the section of the wire, as for example for higher sub-bands.

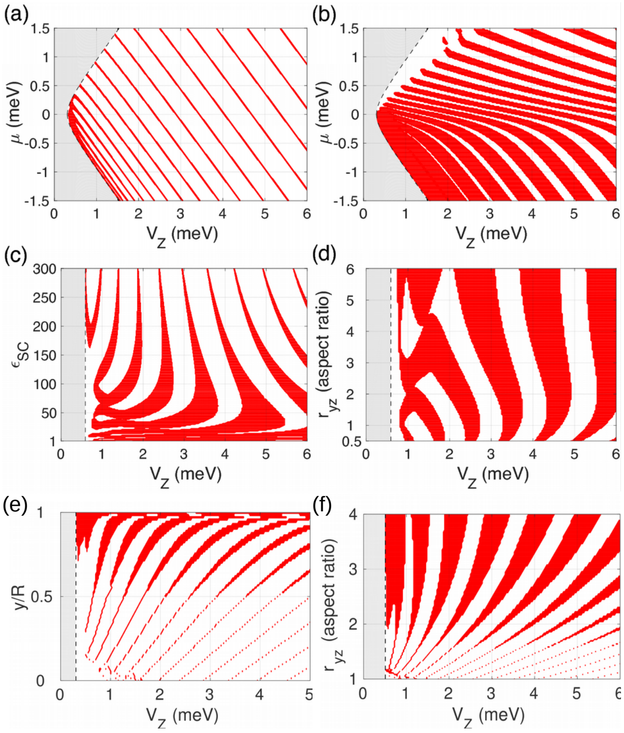

Figure S4: Phase diagrams indicating the parameter regions where the Majorana bound states are pinned to zero-energy (in red). In the upper panels the phase diagram is calculated as a function of Zeeman field and chemical potential for the non-interacting case (a) and interacting case (without leads) with $\varepsilon_{SC} = 100$ (b). The central panels correspond both to the interacting case, but (c) considers variations in the effective dielectric constant of the thin superconducting layer, ε_{SC}, whereas (d) explores different aspect ratios of the nanowire's section $r_{yz} = W_y/W_z$, where $W_{y,z}$ are the y and z widths of the nanowire faces. In the lower panels we consider perfect screening by the SC shell, $\varepsilon_{SC} \to \infty$, but we vary the distance between the transversal Majorana charge density and the SC shell. In (e) y/R is the position of the Majorana wave function across the nanowire section. When $y = 0$ the charge density is at the center of the nanowire, whereas when $y = R$ it is a the opposite face of the SC. In (f) the wave function is fixed at the center of the nanowire section, but we vary its aspect ratio. Here $\mu = 0.5$meV and $W_z = 100$nm, as in the main text.

References

1. Reitz, J. R.; Milford, F. J.; Chrysty, R. W. *Foundations of Electromagnetic Theory*, 4th ed.; Addison-Wesley Pub. Co., 1997.

2. Domínguez, F.; Cayao, J.; San-Jose, P.; Aguado, R.; Yeyati, A. L.; Prada, E. *npj Quantum Mater.* **2017**, *2*, 13. doi:10.1038/s41535-017-0012-0.

3. Giuliani, G.; Vignale, G. *Quantum Theory of the Electron Liquid*, 1st ed.; Cambridge University Press, 2005.

4. Vuik, A.; Eeltink, D.; Akhmerov, A. R.; Wimmer, M. *New J. Phys.* **2016**, *18*, 033013. doi: 10.1088/1367-2630/18/3/033013.

5. Knapp, C.; Karzig, T.; Lutchyn, R.; Nayak, C. *Phys. Rev. B* **2018**, *97,* 125404.
